# Contribution of *GalU* to biofilm formation, motility, antibiotic and serum resistance, and pathogenicity of *Salmonella* Typhimurium

**DOI:** 10.3389/fcimb.2023.1149541

**Published:** 2023-03-20

**Authors:** Lili Guo, Huilin Dai, Saixiang Feng, Yongda Zhao

**Affiliations:** ^1^ College of Veterinary Medicine, Qingdao Agricultural University, Qingdao, China; ^2^ College of Veterinary Medicine, South China Agricultural University, Guangzhou, China

**Keywords:** *Salmonella* Typhimurium, *galU*, biological characteristics, virulence pathogenicity, biofilm

## Abstract

**Introduction:**

*Salmonella* Typhimurium is the leading cause of foodborne illnesses in China, resulting in major epidemics and economic losses in recent years. Uridine diphosphate–glucose pyrophosphorylase *galU* plays an important role in thebiosynthesis of the bacterial envelope. Herein, we evaluated the role of *galU* in *S*. Typhimurium infection in chicken.

**Methods:**

A *galU* gene mutant was successfully constructed by red homologous recombination technology, and biological characteristics were studied.

**Results:**

The *galU* mutant strain had a rough phenotype;was defective in biofilm formation, autoagglutination, and motility; exhibited greater sensitivity to most antibiotics, serum, and egg albumen; and had lowercapacity for adhesion to chicken embryo fibroblasts cell line (DF-1). The *galU* mutant showed dramatically attenuated pathogenicity in chicken embryos (100,000-fold), BALB/c mice (420-fold), and chicks (100-fold).

**Discussion:**

The results imply that *galU* is an important virulence factor in the pathogenicity of *S.* Typhimurium, and it may serve a target for the development of veterinary drugs, providing a theoretical basis for the prevention and control of *S.* Typhimurium.

## Introduction

*Salmonella enterica* is a highly diverse Gram-negative bacterium, a major public health concern, and it affects the health of animals (Huang et al., 2019). Over 2,600 serotypes have been identified worldwide, of which *S.* Typhimurium is among the most frequent. It has been isolated from humans, animals, food, and the wider environment, it accounts for ~94 million cases of self-limiting gastroenteritis in humans annually, and it causes diarrhea in livestock (Huang et al., 2019; Aguilera et al., 2022). *S.* Typhimurium is a major cause of high mortality in poultry farming; it has led to significant economic losses in the poultry industry worldwide (Lacharme-Lora et al., 2019). The complement system is an important part of innate immunity in animals and the first line of defense. Complement activation triggers a series of reactions that lead to the formation of membrane attack complexes (MACs), resulting in the lysis or death of pathogens (Walport, 2001). The complement sensitivity of *S.* Typhimurium strains from different clinical sources vary widely.

Uridine diphosphate–glucose pyrophosphatase *galU* is essential for the production of UDP-glucose and required for the synthesis of lipopolysaccharides (LPSs). UDP-glucose as a substrate is involved in the biosynthesis of capsular polysaccharides that can promote complement resistance and extracellular polysaccharides that participate in biological membrane formation (Nesper et al., 2001; Zou et al., 2013; Guo et al., 2017). Studies have reported that *galU* is essential for the synthesis of the surface structures of a variety of virulence-related factors that lead to bacterial attachment, motility, colonization, biofilm formation, serum sensitivity, and the attenuation of virulence in many Gram-negative pathogens (Chang et al., 1996; Vilches et al., 2007; Jayakar et al., 2011; Zou et al., 2013; Meyer et al., 2015; Zhao et al., 2021; Shu et al., 2022). To date, the genome sequences of some *S.* Typhimurium strains have been reported, and some virulence factors involved in immune responses have been identified (Das and Sharma, 2018; Ben et al., 2021). However, the role of *galU* in *S.* Typhimurium remains unclear, and the relationship between *galU* and virulence in *S.* Typhimurium is poorly understood.

In the current study, we explored the biological characteristics of *galU* in *S.* Typhimurium in terms of autoagglutination and motility, cell adhesion ability, antibiotics and serum sensitivity, and virulence in animal models. Additionally, we investigated the relationship between *galU* and pathogenicity and attempted to identify novel drug targets for disease therapy.

## Materials and methods

### Ethics statement

All animal experiments conducted in this study were approved by the Animal Ethics Committee of Qingdao Agricultural University (No. 2020-025). All procedures involved were designed and performed in accordance with the guidelines issued by this committee to minimize animal suffering and maximize animal welfare.

### Bacteria, cells, specific pathogen free (SPF) chicken embryos, chicks, and BALB/c mice

*Escherichia coli* WM3064 cells were grown in Luria–Bertani (LB) broth at 37°C. *S.* Typhimurium strain S584 isolated from chickens from China was stored in our laboratory and cultivated in LB at 37°C. Plasmids pKD46 (Datsenko and Wanner, 2000), pCP20, and pBBR1MCS2 (Kovach et al., 1995) were used in this study. DF-1 cells were prepared from SPF chicken embryos (Meria, Beijing, China) and cultured in Dulbecco’s modified Eagle’s medium (DMEM) containing 10% fetal bovine serum at 37°C in 5% CO_2_. SPF chicken embryos and SPF chicks were purchased from Meria and fed in our animal room. BALB/c mice were purchased from Qingdao Dawu Fucheng Technology Co. (Qingdao, China).

### Mutation and complementation of *galU*

#### Mutation strain construction

All primers involved in this study are detailed in **Supplementary Table 1**. A DNA fragment of 1,567 bp was PCR-amplified using primers *galU*-Q. The electrotransformation-receptive cells of the S584 strain were prepared. The pKD46 plasmid was transformed into S584-receptive cells to obtain S584/pKD46 cells. The target fragment of the *galU* gene was directly transformed into the receptive cells. The cells were plated on an LB agar medium containing kanamycin (Kn; 30 μg/ml) and cultured overnight at 37°C. PCR amplification using primers *galU*-Kn was used to screen positive clones whose *galU* gene was replaced by the Kn resistance gene, named S584/Δ*galU*-Kn. The electrotransformation-receptive cells of S584/Δ*galU*-Kn were prepared and transformed with plasmid pCP20 to obtain S584/Δ*galU*-Kn/pCP20. Following the mutation of Kn and pCP20, S584/Δ*galU*-KN/pCP20 cells were cultured at 42°C to obtain the *galU* mutant, identified by the *galU*-JD primer, named S584/Δ*galU*.

#### Complement strain construction

The *galU* fragment was PCR-amplified using the *galU*-HF primer and cloned into the *EcoRV* site of the pBBR1MCS2 plasmid *via* the In-fusion method. After transformation, positive clones were screened on LB plates containing Kn. The electrotransformation-receptive cells of *E. coli* strain WM3064 were prepared. After melting cells in an ice bath, 10 ng purified pBBR1MCS2-*galU* plasmid was added. After electrotransformation, transformed bacteria were immediately suspended in 1 ml of fresh LB containing 0.5 mM diaminoheptadecanoic acid (DAP; Aladdin reagent) and agitated at 220 rpm and 37°C for 1 h. A 50 μl volume of cells was spread on an LB plate containing 0.5 mM DAP and 50 μg/ml Kn and cultured at 37°C until colony formation. Recipient bacteria S584Δ*galU* were inoculated on an LB plate *via* streaked lines and cultured at 37°C until colony formation. Single clones were cultured in 3 ml of an LB medium with shaking at 220 rpm overnight. A WM3064/pBBR1MCS2-*galU* clone was selected from the plate and placed into 3 ml of LB (containing 50 μg/ml Kn and 0.5 mM DAP) and cultured overnight at 220 rpm and 37°C as donor bacteria. A 500 μl volume of donor bacteria WM3064/pBBR1MCS2-*galU* was mixed with 500 μl of recipient bacteria in the conjugation experiment. A 100 μl volume of the conjugation sample was spread on an LB plate containing Kn (50 μg/ml) and cultured at 30°C until colony formation. A well-grown single clone was selected and identified by the pBBR1 primer as the *galU* gene complement strain S584Δ*galU* R.

### Motility test

Cell motility was tested as described previously (Gomez-Baltazar et al., 2019). *S.* Typhimurium strains were cultured at 37°C overnight; 100 μl of culture was inoculated onto the center of a 0.3% LB agar plate and incubated at 37°C for 8 or 24 h. The diameter moved by the three strains was measured and averaged from three replicates.

### Rough phenotypic identification

The Acridine Orange agglutination test and *Salmonella* O_9_ standard serum agglutination test were performed as described previously (Guo et al., 2019) to explore the rough colony phenotypic characteristics of the *S.* Typhimurium strains. A 20 µl volume of *Salmonella* O_9_ serum and 0.2% Acridine Orange were applied separately to clean slides, and the same amount of bacterial suspension was aseptically absorbed and mixed with them. Normal saline served as a negative control, and samples were incubated at room temperature for 1−2 min. Agglutination reactions were observed under good illumination, and reactions with agglutination particles were judged as positive, while those with no agglutination particles were judged as negative.

### Autoagglutination assay

The autoagglutination testing of *S.* Typhimurium strains was performed as previous described (Zhou et al., 2014) with some modifications. Specifically, *S.* Typhimurium strains were cultured in LB broth overnight, 0.5 ml of the upper bacterial liquid close to the liquid level was carefully absorbed, and the OD_600_ value (OD_600_Pre) was measured. The remaining liquid was vortexed vigorously, and 0.5 ml was taken to measure OD_600_ (OD_600_Post). The following formula was used to evaluate the self-agglutination ability: self-agglutination rate (%) = [(OD_600_Post - OD_600_Pre)/OD_600_Post] ×100%.

### Biofilm formation assay

Biofilm morphology was observed by confocal laser scanning microscopy according to a previous protocol (Xie et al., 2016). Bacterial cultures (S584, S584Δ*galU*, and S584Δ*galU* R strains) grown overnight at 28°C for 24 h in a confocal laser culture dish were washed three times with sterilized phosphate-buffered saline (PBS), 1 ml of 2.5% glutaraldehyde was added and incubated for 30 min, and samples were washed three times with sterile PBS. After drying at room temperature, staining with 4',6-diamidino-2-phenylindole (DAPI) nucleic acid dye and incubating in the dark for 15 min, samples were rinsed three times with sterile PBS. In accordance with the procedures for the laser confocal scanning microscope, biofilms were observed under fluorescence mode.

Biofilm formation (ocular view) was assessed using a previously described method (Zou et al., 2013) with slight modifications. Briefly, 10 µl of bacterial culture was added to borosilicate glass tubes containing 4 ml of LB and incubated for 24 h at 28°C without agitation. The suspensions were removed by a pipette and stained with 5 ml of Crystal Violet solution for 5 min. The dye solution was removed, samples were washed thoroughly under tap water for at least 3 min, and tubes were inverted on a paper towel to remove excess water and then photographed.

Biofilm quantification testing was performed as previously described (Gomez-Baltazar et al., 2019). Briefly, S584, S584Δ*galU*, and S584Δ*galU* R strains were cultured by inoculating into a 96-well sterile polystyrene microtiter plate, incubating at 28°C for 48 h, and non-adhered cells were removed. Glacial acetic acid was added to the sterile polystyrene microtiter plate after ethanol fixation and the Crystal Violet staining of adhered biofilms. The absorbance was determined at 595 nm using a plate reader. Each strain was tested in three independent experiments.

### Antimicrobial susceptibility assay

Antimicrobial susceptibility testing was performed using the agar dilution method. The S584, S584Δ*galU*, and S584Δ*galU* R strains were tested for susceptibility to 25 antimicrobial agents including nalidixic acid, levofloxacin, norfloxacin, enrofloxacin, lomefloxacin, ciprofloxacin, meroxacin, aztreonam, cefepime, cefotaxime, ceftriaxone, ampicillin, azithromycin, tylosin, tildipirosin, chloramphenicol, doxycycline, tetracyclic, apramycin, streptomycin, gentamicin, spectinomycin, amikacin, sulfamethoxazole, and polymyxin E. *Escherichia coli* strain ATCC 25922 was used as a quality control strain. Minimum inhibitory concentration (MIC) was determined by referring to standards from the Clinical and Laboratory Standards Institute (CLSI) documents M100-S28.

### Serum sensitivity assay

Serum sensitivity tests were performed using a previously described method (Zou et al., 2013; Guo et al., 2017) with slight modifications. Briefly, sera were collected from SPF chickens (3 weeks of age), filter-sterilized (0.22 μm), and stored at -80°C. Prior to testing, SFP chicken serum was treated at 56°C for 30 min to inactivate the complement cascade. A 100 µl volume of an overnight culture of bacteria (1 × 10^8^ Colony Forming Units (CFU)/ml) was added to 100 µl of heat-treated serum mixed, and culturing was continued for 1 h at 37°C (with moderate shaking), and culturing was continued for 1 h at 37°C (with moderate shaking), then the mixture was serially diluted in PBS for live bacteria counting.

### Survival in egg albumen

The survival ability in egg albumen was assayed as previously described (Guo et al., 2017). Antibiotic-free eggs were aseptically broken and collected in a sterile container. A 1 ml volume of bacterial cultures (1 × 10^8^ CFU/ml) was mixed with a 1 ml aliquot of egg albumen and incubated at 37°C for 24 h after thorough mixing. The mixtures were serially diluted in PBS for live bacteria counting. Each strain was tested in three independent experiments.

### Adherence assay

Chicken embryonic fibroblast DF-1 cells were used for the bacterial adhesion assay as described previously (Mills and Finlay, 1994; Guo et al., 2017) with slight modifications. Briefly, DF-1 cells were prepared, added to DMEM containing 10% fetal bovine serum, and cultured at 37°C and 5% CO_2_. After culturing for 24 h, DF-1 cells were infected with fresh overnight cultures and incubation was continued for 2 h. Cells were washed to remove non-adhered bacteria and lysed with 0.1% Triton X-100, and bacterial counting was performed on LB agar plates. Non-infected DF-1 cells were used as a negative control, and the experiment was repeated three times.

### Pathogenicity assay

#### Pathogenicity toward SPF chicken embryos

Pathogenicity toward SPF chicken embryos was assessed as described previously (Guo et al., 2017). The lethality of the three strains (S584, S584Δ*galU*, and S584Δ*galU* R) against chicken embryos was determined. A total of 310 11-day-old chicken embryos were divided into 31 groups with 10 eggs per group. The bacterial solution was serially diluted 10-fold from 10^10^ to 10 CFU/ml. Allantois cavity inoculation was performed. In groups 1–10, each chicken embryo was inoculated with 0.1 ml of S584 (1 × CFU/egg−1 × 10^9^ CFU/egg). In groups 11–20, each chicken embryo was inoculated with 0.1 ml of S584Δ*galU* (1 × CFU/egg−1 × 10^9^ CFU/egg). In groups 20–30, each chicken embryo was inoculated with 0.1 ml of S584Δ*galU* R (1 × CFU/egg−1 × 10^9^ CFU/egg). In group 31, each embryo was inoculated with sterile PBS as a negative control. Inoculation sites were sealed with paraffin wax, and all chicken embryos were incubated at 37°C with 50%−60% relative humidity. After 72 h of incubation, the viability and mortality of chicken embryos was checked by candlelight, according to the integrity of the venous system and the movement of embryos. The 50% lethal dose of eggs (ELD_50_) was calculated.

#### Pathogenicity toward BALB/c mouse

A total of 100 BALB/c mice were randomly divided into 10 groups, with 10 mice per group. Groups 1–3 received 0.2 ml of S584Δ*galU* (10^8^, 10^9^, or 10^10^ CFU), groups 4–6 received 0.2 ml of S584 (10^7^, 10^8^, or 10^9^ CFU), group 7–9 received 0.2 ml of S584Δ*galU* R (10^7^, 10^8^, or 10^9^ CFU), group 10 received 0.2 ml of PBS as a blank control. At 14 days after challenge, the morbidity and mortality of mice were observed and recorded daily. The Bliss method (Yang et al., 2022) was used to calculate the 50% lethal dose (LD_50_).

#### Pathogenicity toward chick

A total of 160 1-day-old SPF chicks were randomly divided into 16 groups, with 10 chicks in each group. Groups 1–5 were injected with 0.2 ml of S584Δ*galU* (10^6^ CFU/egg−10^10^ CFU/egg); group 6–10 were injected with 0.2 ml of S584 (10^6^ CFU/egg−10^10^ CFU/egg); group 11–15 were injected with 0.2 ml of S584Δ*galU* R (10^6^ CFU/egg−10^10^ CFU/egg); group 16 was injected with 0.2 ml of PBS as blank control. At 14 days after challenge, the morbidity and mortality of chicks were observed and recorded daily. The Bliss method (Yang et al., 2022) was used to calculate LD_50_.

### Statistical analysis

Statistical analysis was performed with Statistics software SPSS version 23.0 (IBM, Chicago, IL, USA), and statistical significance was established at a *p* < 0.05 (Feng et al., 2020).

## Results

### Construction and characterization of *galU* mutants

**Figure 1:**
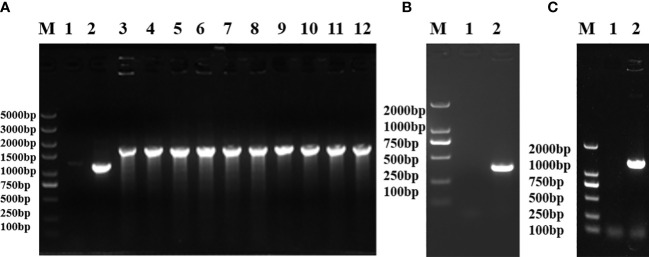
Identification of uridine diphosphate–glucose pyrophosphorylase *galU* gene mutants. **(A)** Replacement of the *galU* gene with the Kn resistance gene. Lane M, molecular mass markers; Lane 1, negative control; Lane 2, S584; Lane 3−12, S584Δ*galU*-Kn. **(B)** Confirmation of *galU* mutation showing gene loss in *S. typhimurium* lacking a Kn resistance gene. Lane M, molecular mass markers; Lane 1, negative control; Lane 2, S584Δ*galU*. **(C)** Identification of the complementation strain showing that the *galU* gene was successfully reintroduced after mutation. Lane M, molecular mass markers; Lane 1, negative control; Lane 2, S584Δ*galU* R.

The electrotransformation-receptive cells of *Salmonella* S584 were prepared, plasmid pKD46 was electrotransferred into S584 cells, and they were cultured overnight at 30°C on a plate containing 100 μg/ml ampicillin. A single S584/pKD46 colony was selected to prepare electrotransformation-receptive cells. For the preparation of the *galU* target fragment, plasmid pKD4 served as a template, and amplification was performed using the pfu enzyme. When the *galU* gene was replaced by the Kn resistance gene, a fragment of 1,786 bp was identified and the strain was named S584Δ *galU*:: Kn (**Figure 1A**). The Kn resistance gene was deleted, and the mutant strain was named S584Δ*galU* (**Figure 1B**). The constructed expression plasmid was electrotransferred into *E. coli*–receptive cells; after conjugation, the complementation strain S584Δ*galU* R was obtained (**Figure 1C**).

### Loss of *galU* enhances autoagglutination and sensitivity to serum and egg albumen

**Figure 2:**
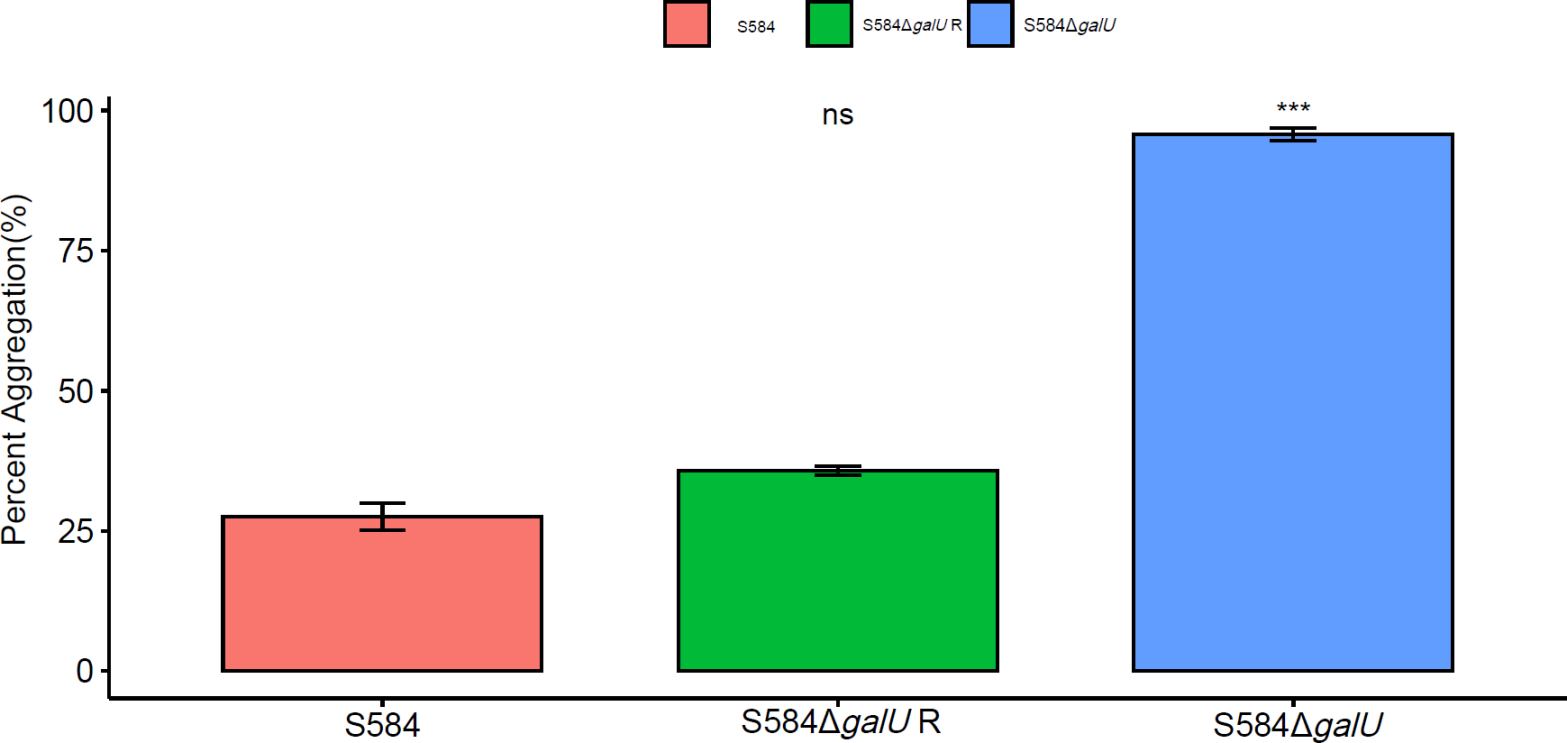
Self-agglutination properties. Different colors represent different strains (red = S584, green = S584Δ*galU* R, and blue = S584Δ*galU*). The vertical coordinate of the graph shows the self-agglutination rate for each strain. Ns means no significant difference. *** means very significant difference.

Agglutination test results showed that S584, S584Δ*galU*, and S584Δ*galU* R showed significant differences in turbidity in LB culture (*p <*0.05). The average self-agglutination rates of S584, S584Δ*galU*, and S584Δ*galU* R strains were 27.54 ± 4.11%, 95.76 ± 1.95%, and 35.77 ± 1.41%, respectively. The self-agglutination rate of strain S584Δ*galU* was 3.47 times faster than that of strain S584, and the difference was extremely significant (*p* < 0.01), but there was no significant difference between the self-agglutination rate of S584 and S584Δ*galU* R strains (*p >* 0.05; **Figure 2**).

**Figure 3:**
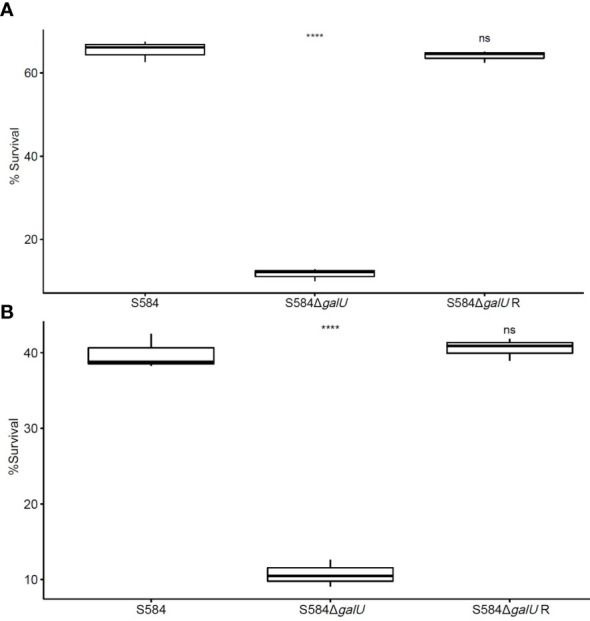
Sensitivity to serum and egg albumen. **(A)** Results of anti-complement-mediated serum killing assays. **(B)** Survival in egg whites. The vertical coordinate of the graph shows the survival rate for each strain in serum or egg albumen. Ns means no significant difference. **** means extremely significant difference.

The results of sensitivity to serum and egg albumen tests showed significant differences between strains; S584Δ*galU* had a survival rate in the serum of 11.68 ± 1.49% (**Figure 3A**), and the survival rate in egg albumen was 10.73 ± 1.78% (**Figure 3B**). By contrast, parent strain S584 was relatively resistant to egg albumen and serum, with the survival rates of 65.42 ± 2.52% in serum and 39.86 ± 2.30% in egg albumen (*p* < 0.01; **Figure 3**).

### Mutation of *galU* may be related to phenotypic characteristic and reduce motility

**Figure 4:**
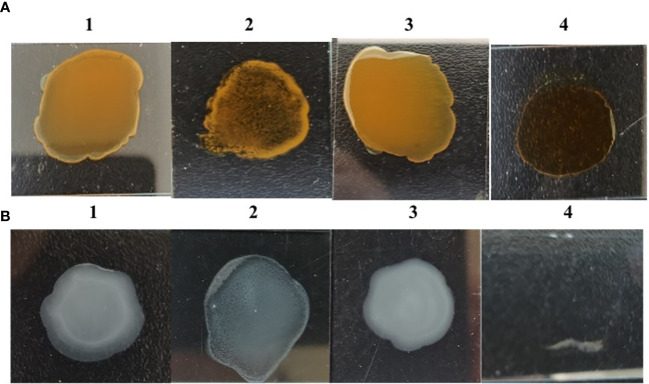
Rough phenotype identification. **(A)** Results of Acridine Orange agglutination tests. 1, parental strain S584; 2, mutation strain S584Δ*galU*; 3, complementation strain S584Δ*galU* R; 4, 0.2% Acridine Orange negative control. **(B)** *Salmonella* O_9_ standard serum agglutination test results. 1, parental strain S584; 2, mutation strain S584Δ*galU*; 3, complementation strain S584Δ*galU* R; and 4, negative control.

The results of rough phenotypic identification showed that S584Δ*galU* underwent an agglutination reaction with the single-factor diagnostic serum of O_9_, but S584 and S584Δ*galU* R strains did not. Strain S584Δ*galU* underwent agglutination with 0.2% Acridine Orange, while S584 and S584Δ*galU* R strains did not (**Figure 4**). These results showed that S584Δ*galU* displayed the characteristics of a rough strain.

**Figure 5:**
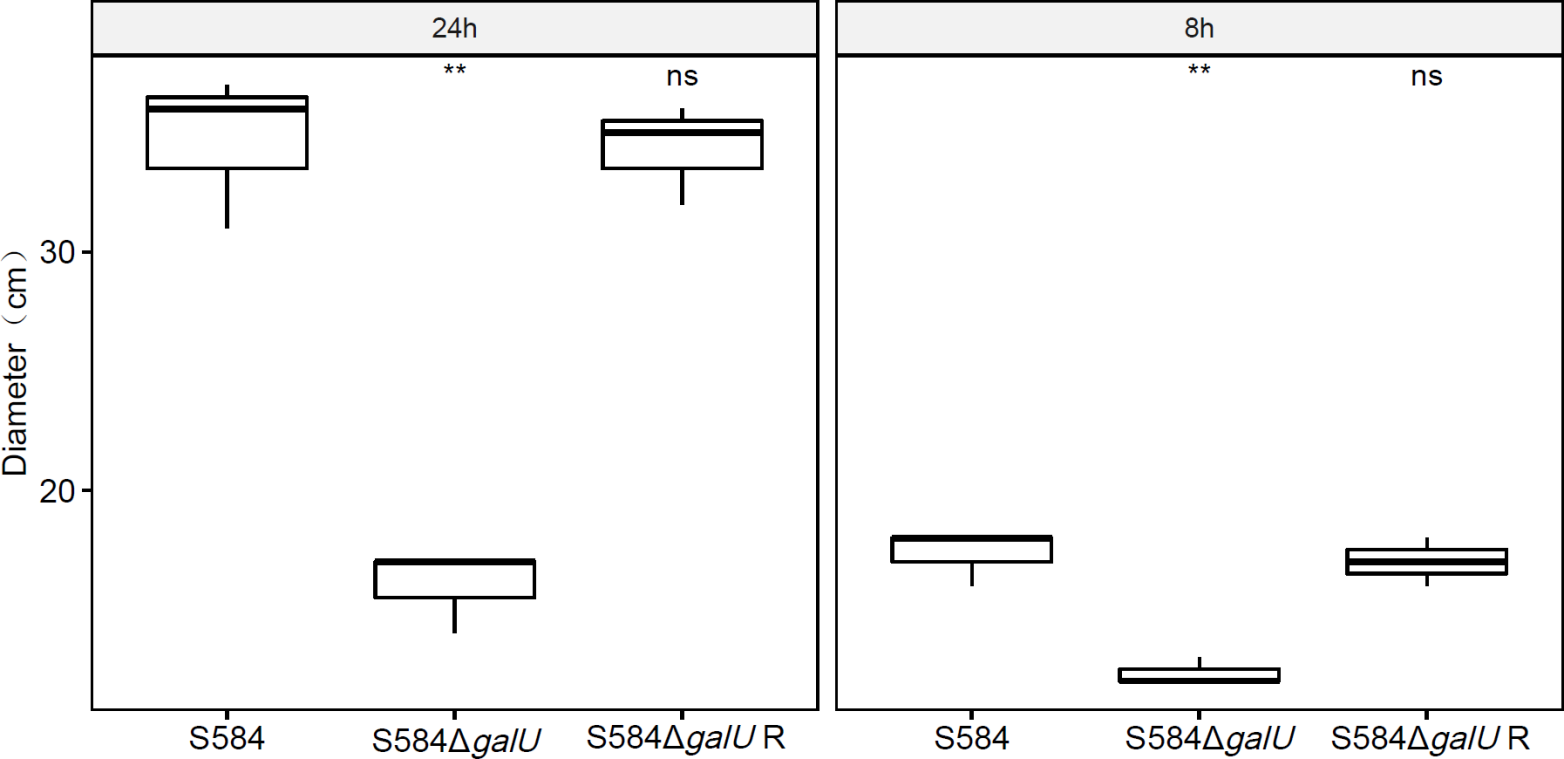
Results of motility analysis for the three strains at 8 and 24 h. The vertical coordinate of the graph shows the movement diameter (cm) for each strain cultured on Luria–Bertani (LB) plates after 8 and 24 h. Ns means no significant difference. ** means significant difference.

After 8 h of culture, S584 had a motion diameter of 17.33 ± 1.15 mm, S584Δ*galU* had a motion diameter of 12.33 ± 0.58 mm, and S584Δ*galU* R had a motion diameter of 17 ± 1 mm. After 24 h of culture, the motion diameter of S584 was 34.67 ± 3.21 mm, that of S584Δ*galU* was 16 ± 1.73 mm, and that of S584Δ*galU* R was 34.33 ± 2.08 mm. Compared with the parent strain, the motor diameter of S584Δ*galU* was decreased by 29.41% (8 h) and 54.29% (24 h). The difference was significant (*p* < 0.01; **Figure 5**).

### Mutation of *galU* causes defective biofilm formation and enhances antibiotic sensitivity

#### Biofilm formation assays

**Figure 6:**
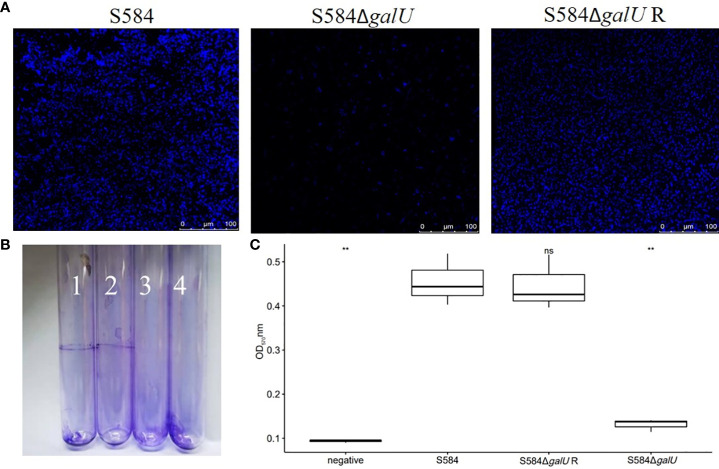
Biofilm formation ability. **(A)** Confocal laser scanning microscopy analysis. **(B)** Photographs of test tubes from binding assays. **(C)** Biofilm production of the three bacterial strains cultured in a 96-well microplate for 48 h. Results are mean ± standard error of the mean (SEM). The bottom dashed line shows the node where the biofilm was measured (ODc = 0.1651). According to the OD value, strains were divided into four categories: non-biofilm producers (OD ≤ODc), weak biofilm producers (ODc <OD ≤2×ODc), medium biofilm producers (2×ODc <OD ≤4×ODc), and strong biofilm producers (2ODc <OD). Ns means no significant difference. ** means significant difference.

The results of confocal laser scanning microscopy showed that the bacterial aggregation morphology of S584 and S584Δ*galU* strains was significantly different. The cell morphology analysis of S584Δ*galU* revealed a scattered state, while the cells of S584 and S584Δ*galU* R displayed a flake stacking distribution (**Figure 6A**). The results of Crystal Violet biofilm staining showed that S584Δ*galU* did not produce biofilm, while S584Δ*galU* R and S584 did produce biofilm (**Figure 6B**). The results of biofilm quantification were similar for S584Δ*galU* R and S584 strains, with medium biofilm-forming ability, but S584Δ*galU* did not produce biofilm (**Figure 6C**). The biofilm formation abilities of S584Δ*galU* and S584 showed significant differences (*p* <0.05).

#### Antimicrobial susceptibility

**Figure 7:**
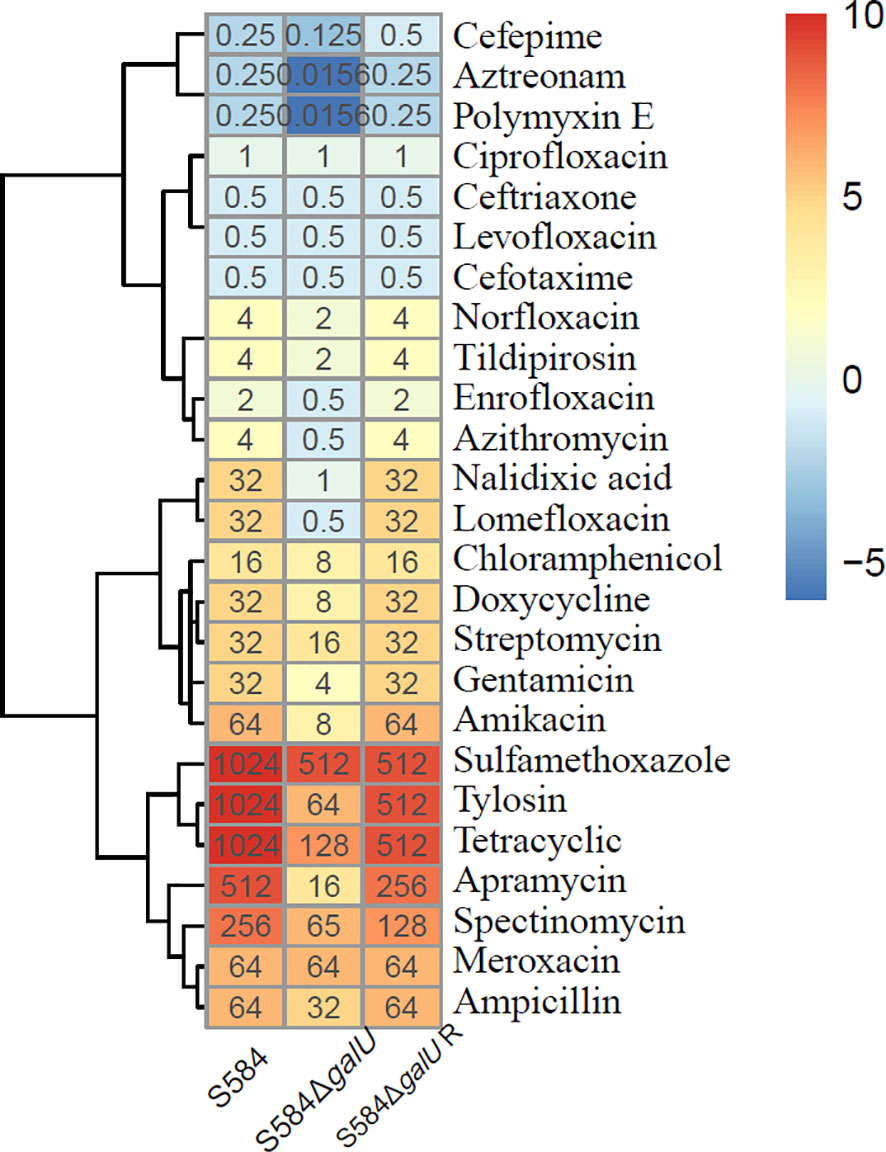
Comparison of antibiotic susceptibility of the three strains. Different colors represent the minimum inhibitory concentration (MIC) values of each strain to antibiotics; the darker the color, the higher the MIC for the antibiotic, indicating a strong resistance to the antibiotic.

The results of the antimicrobial susceptibility analysis showed that S584Δ*galU* was sensitive to most antibiotics (**Figure 7**). After the mutant of *galU*, the isolate showed a 2- to 64-fold decrease in quinolone MIC, a 2- to 16-fold decrease in β-lactam MIC, a 2- to 16-fold decrease in macrolide MIC, a 2-fold decrease in chloramphenicol MIC, a 4- to 8-fold decrease in tetracycline MIC, a 2- to 32-fold decrease in aminoglycoside MIC, a 2-fold decrease in sulfamethoxazole MIC, and a 16-fold decrease in polymyxin E MIC. Notably, S584ΔgalU showed a 16-fold reduction in resistance to aztreonam, tylosin, and polymyxin E, a 32-fold reduction in resistance to nalidixic acid and apramycin, and a 64-fold reduction in resistance to lomefloxacin. However, the MICs for five antibiotics (levofloxacin, ciprofloxacin, meroxacin, cefotaxime, and ceftriaxone) did not differ between S584 and S584ΔgalU strains. Antibiotic resistance was not significantly different between S584ΔgalU R and S584 strains (**Figure 7**).

### Mutation of *galU* decreases cell adhesion capacity

**Figure 8:**
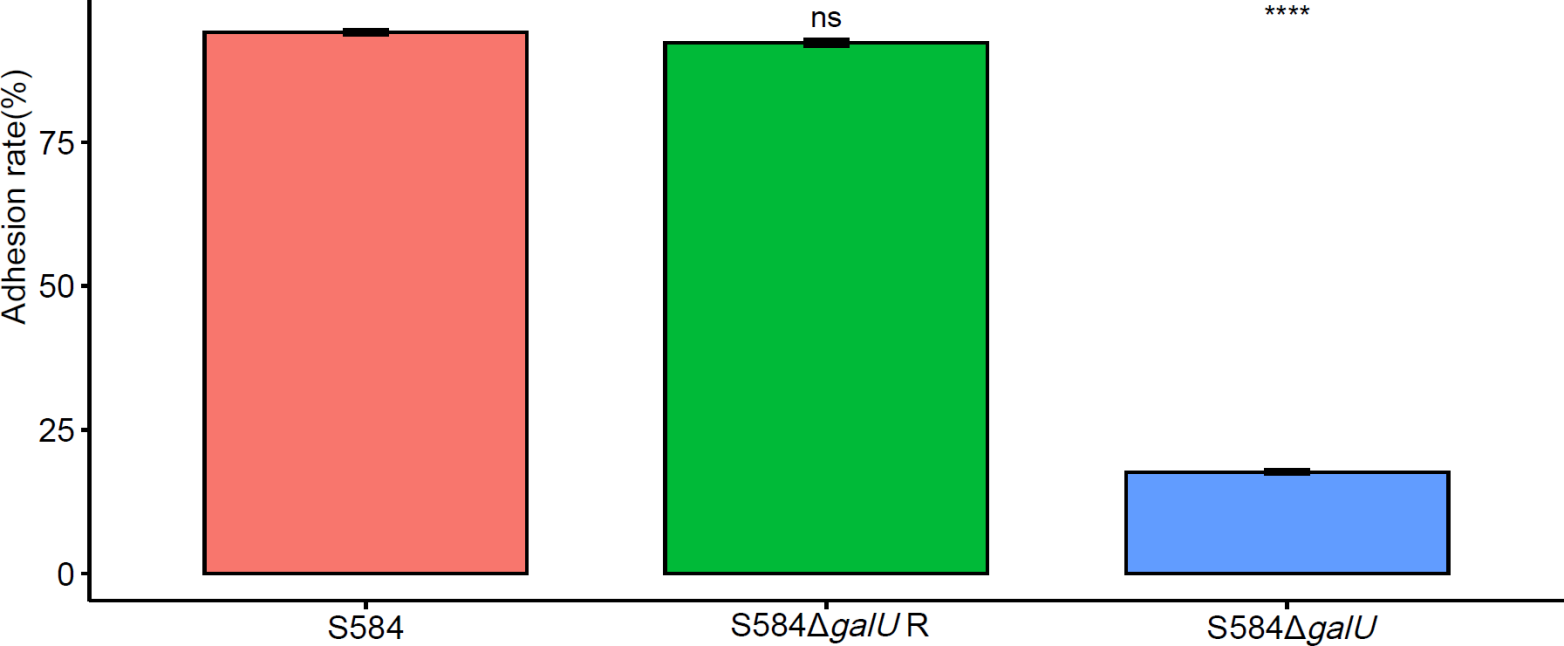
Adhesion ability of the three bacterial strains to DF-1 cells. Different colors represent different strains (red = S584, green = S584 Δ*galU* R, and blue = S584 Δ*galU*). The vertical coordinate of the graph shows the adhesion rate for each strain to DF-1 cells. Ns means no significant difference. **** means extremely significant difference.

The results of the adhesion testing of Salmonella to DF-1 cells showed that the adhesion rate of S584 to DF-1 cells was 94.16 ± 0.75%, compared with 17.65 ± 0.58% for S584Δ*galU*, a significant reduction of 81.26% (*p* < 0.05). These results demonstrate that, after *galU* gene mutation, the ability to adhere to cells was decreased, indicating a close association with cell adhesion (**Figure 8**).

### *GalU* is an important pathogenic factor in *S.* Typhimurium

#### Pathogenicity toward SPF chicken embryos

**Figure 9:**
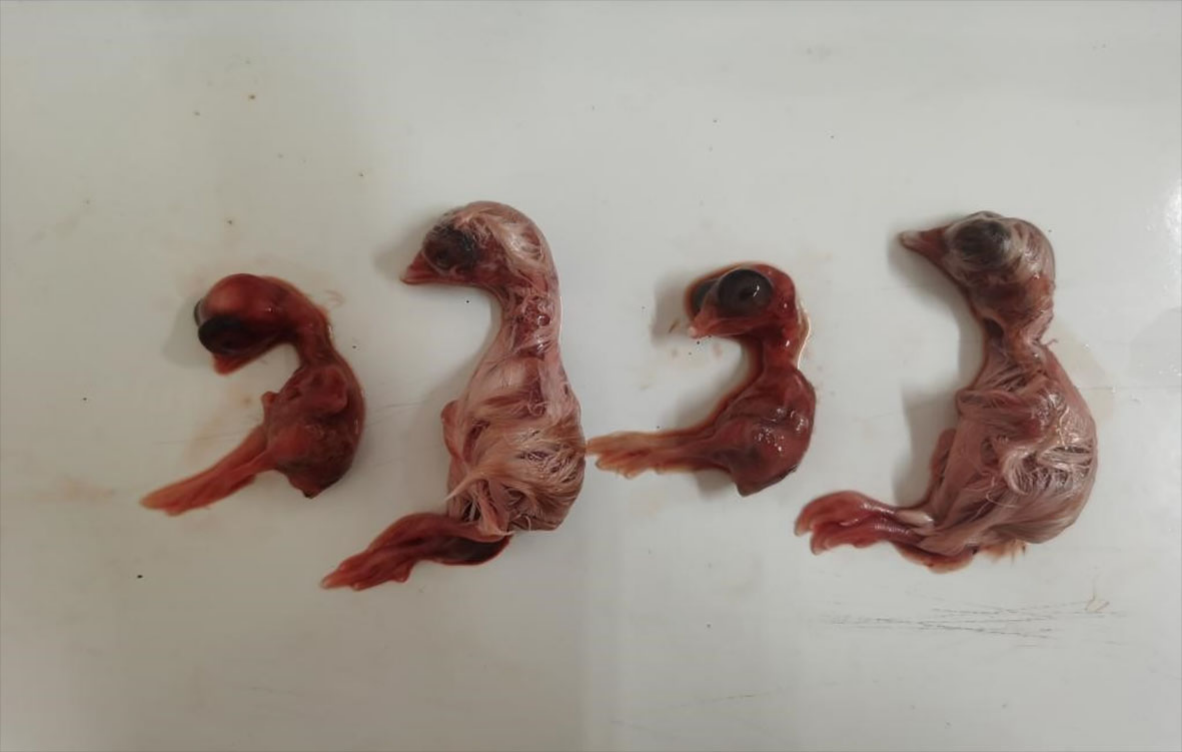
Pathogenicity in chicken embryos (1 × 10^3^ CFU). Chicken embryos are ordered S584, S584Δ*galU*, S584Δ*galU* R, and control strains from left to right.

The results showed that, in the S584 strain group, the mortality rate of chicken embryos inoculated with 10^3^ CFU was 100%, and the ELD_50_ was 6.8×10^3^ CFU (**Table 1**). In the S584Δ*galU* strain group, the mortality rate of chicken embryos inoculated with 10^8^CFU was 100%, and the ELD_50_ was 6.8 × 10^8^ CFU (**Table 1**). In the S584Δ*galU* R strain group, the mortality rate of chicken embryos inoculated with 10^3^ CFU was 100% and the ELD_50_ was 5.1×10^3^ CFU (**Table 1**). According to the ELD_50_ results, the pathogenicity of S584 toward chicken embryos was 100,000 times that of S584Δ*galU* (**Table 1**). The somatic lesions of chicken embryos inoculated with strain S584 or S584Δ*galU* R inoculated with 10^3^ CFU showed obvious lesions, including multifocal skin bleeding and subcutaneous edema. However, no lesions were found in the chicken embryos of the S584Δ*galU* or PBS control groups (**Figure 9**). These results indicate that *galU* has a strong influence on pathogenicity toward chicken embryos.

**Table 1:** Virulence analysis of three strains in chicken embryos.

Group	Death number										ELD_50_
	1	10^1^	10^2^	10^3^	10^4^	10^5^	10^6^	10^7^	10^8^	10^9^	
S584	1	1	4	10	10	10	10	10	10	10	6.8 × 10^3^
S584Δ*galU*	0	0	0	0	0	0	0	4	10	10	6.8 × 10^8^
S584Δ*galU* R	0	1	3	10	10	10	10	10	10	10	5.1 × 10^3^
Control group	0	/	/	/	/	/	/	/	/	/	/

The / symbol indicates that this part of the test was not carried out.

#### Pathogenicity toward BALB/c mice

The results showed that the LD_50_ values for S584, S584Δ*galU*, and S584Δ*galU* R strains were 1.6 × 10^8^, 6.8 × 10^10^, and 1.9 × 10^8^ CFU, respectively (**Table 2**). These results showed that the LD_50_ value was increased ~420-fold after *galU* gene mutation.

**Table 2:** Virulence analysis of three strains in BALB/c mice.

Group	Death number				LD_50_
	10^7^ CFU	10^8^ CFU	10^9^ CFU	10^10^ CFU	
S584	1	6	10	/	1.6 × 10^8^
S584Δ*galU*	/	0	4	10	6.8 × 10^10^
S584Δ*galU* R	0	7	10	/	1.9 × 10^8^
Control group	0	0	0	0	/

The / symbol indicates that this part of the test was not carried out.

#### Pathogenicity toward chicks

The results showed that the LD_50_ values for S584, S584Δ*galU*, and S584Δ*galU* R strains were 1.7 × 10^8^, 1.4 × 10^10^, and 2.5 × 10^9^ CFU, respectively, and all chicks in the negative control group survived (**Table 3**). These results showed that the LD_50_ values of chicks was increased ~100-fold after *galU* gene mutation, indicating that *galU* had a strong influence on the pathogenicity of chicks.

**Table 3:** Virulence analysis of three strains in chicks.

Group	Death number					LD_50_
	10^6^ CFU	10^7^ CFU	10^8^ CFU	10^9^ CFU	10^10^ CFU	
S584	0	2	6	9	10	1.7 × 10^8^
S584Δ*galU*	0	0	0	0	6	1.4 × 10^10^
S584Δ*galU* R	0	2	7	8	10	2.5 × 10^9^
Control group	0	/	/	/	/	/

The / symbol indicates that this part of the test was not carried out.

## Discussion

Many virulence factors in *Salmonella* have been identified including SPI2, *spiC*, *crp*, *cpxR*, *lon*, *asd*, and *srfA* (Zhang et al., 2020; Ben et al., 2021). The *galU* gene encodes UTP-glucose-1-phosphouridine transferase (UDP glucose-pyrophosphatase), which acts on glucose-1-phosphate and UTP to form UDP-glucose and plays an important role in the biosynthesis of cell envelope–associated carbohydrates (such as LPSs and capsules) in a variety of bacteria (Sandlin et al., 1995; Chang et al., 1996).

There have been many reports on the functions of *galU* in animal pathogens. In *Enterococcus* strains, *galU* is essential for galactose fermentation and the synthesis of polysaccharide antigens that are essential for maintaining normal cell morphology and sensitivity to antimicrobials. The Loss of the *galU* gene attenuated the locomotor ability of *Salmonella* Typhi (Zhao et al., 2021). Consistent with previous results, in the present study, the mutation of *galU* decreased the motor diameter by 29.41% (8 h) and 54.29% (24 h), and the MICs of most antimicrobials were decreased, indicating that *galU* deficiency greatly reduced bacterial motility and increased sensitivity to most antibiotics.

The mutation of *galU* in *Pseudomonas aeruginosa* resulted in the deficiency of the O antigen polysaccharide and phage receptor and enhanced sensitivity to fluoroquinolones and phage, suggesting that studying this gene could contribute to potential strategies for controlling and preventing phage resistance variants in phage therapy (Nakamura et al., 2021). Many studies reported that *galU* is related to cell adhesion capacity and pathogenicity; for example, the mutation of *galU* in *Francisella tularensis* (FT) led to a highly cytotoxic phenotype and rapid induction of IL-1b production after infection *in vitro* and *in vivo*, suggesting that *galU* mutants promote protective immunity against wild-type FT in a sublethal infection in mice (Jayakar et al., 2011). *GalU* mutation in *Shigella* resulted in decreased virulence and decreased invasiveness toward HeLa cells, suggesting that GalU plays a vital role in the pathogenesis of *Shigella* (Sandlin et al., 1995). In *Aeromonas thermophila*, *galU* mutation reduced the survival rate in serum, reduced adhesion, and reduced virulence by 1.5 or 2 logarithmic units in fish or mouse septicemia models (Vilches et al., 2007). The *P. aeruginosa galU* mutant was more sensitive to serum and remained cytotoxic *in vitro* (Priebe et al., 2004). Similarly, in our current work, the *galU* mutant exhibited significantly more sensitivity to serum, and it was less able to adhere to DF-1 cells. It is worth noting that the deficiency of the *galU* mutant drastically attenuated the pathogenicity of *S.* Typhimurium in chicken embryos (decreased by 100,000 times), BALB/c mice (decreased by 420 times), and chicks (decreased by 100 times). These results confirmed that GalU is related to virulence. *GalU* was found to be mainly related to polysaccharide synthesis in *H. parasuis*, involved in the anti-complement-mediated serum killing effect, and the mutant was highly sensitive to serum complement because the synthesis of capsule polysaccharide and LPS was affected (Zou et al., 2013). In our current study, the disruption of *galU* greatly enhanced sensitivity to serum. In *Klebsiella pneumoniae*, after *galU* mutation, bacterial phenotypes including the capsule, LPSs, and several outer membrane proteins were highly sensitive to human serum (Chang et al., 1996). Studies in *Yersinia* plague showed that *galU* mutant strains have increased sensitivity to colistin and catheterin-associated antimicrobial peptides (CRAMPs), and the core of LPS (LOS) is blocked, thereby reducing the ability of bacteria to survive in mouse macrophages (Jayakar et al., 2011).

In our current study, other roles for *galU* related to phenotypic characteristics were evident, including enhanced autoagglutination and sensitivity to egg albumen. However, in previous reports, the *galU* gene was found to be highly polymorphic in *Streptococcus pneumoniae*, which is suitable for molecular typing and phylogenetic studies (Mollerach and García, 2000). In *Lactococcus lactis*, GalU activity levels control intracellular UDP-glucose and UDP-galactose (Boels et al., 2001). In non-pathogenic *E. coli*, the mutation of *galU* altered the LPS O-polysaccharide side chain of biosynthesis, leading to a significant increase in cytokine responses and regulation by macrophage tumor necrosis factor-α (TNF-α) (Meyer et al., 2015), seriously affecting the formation of flagellin in *E. coli* (Komeda et al., 1977). In the present study, after *galU* mutation, the biofilm formation ability, the degree of aggregation of colonies, and cell motility were decreased, while sensitivity to serum and egg albumen was enhanced, and adhesion to DF-1 cells and pathogenic ability were decreased in *S.* Typhimurium. These results are similar to those reported previously for *galU* (Sandlin et al., 1995; Chang et al., 1996; Mollerach and García, 2000; Priebe et al., 2004; Vilches et al., 2007; Jayakar et al., 2011; Zou et al., 2013; Nakamura et al., 2021; Zhao et al., 2021). These results indicate that *galU* is involved in polysaccharide and LPS synthesis, and it plays an important role in the pathogenicity of *S.* Typhimurium.

Notably, this study showed that lack of *galU* in *S.* Typhimurium enhanced the sensitivity to most antibiotics. In addition, the virulence of *S.* Typhimurium was greatly reduced, which provides a new angle for the prevention and control of *S.* Typhimurium, and lays a foundation for studying live vaccine with gene mutation. The findings also provide a theoretical basis for the discovery of new targets related to antibiotic resistance.

## Conclusion

In summary, our results provide insight into the roles of the *galU* gene in *S.* Typhimurium. *GalU* plays an important role in autoagglutination, biofilm formation, antibiotic sensitivity, serum and egg albumen sensitivity, cell adhesion ability, and pathogenicity in *S.* Typhimurium from chicken. This study revealed that *galU* is an important pathogenic factor, expands our understanding of its functions and mechanisms, and may facilitate new technologies for the prevention and control of *S.* Typhimurium disease.

## Data availability statement

The original contributions presented in the study are included in the article/**Supplementary Material**. Further inquiries can be directed to the corresponding authors.

## Ethics statement

The animal study was reviewed and approved by Animal Ethics Committee of Qingdao Agricultural University.

## Author contributions

LG: performing experiments, data analysis, and writing the manuscript. HD: performing experiments. SF: funding acquisition and data analysis. YZ: funding acquisition and data analysis. All authors contributed to the article and approved the submitted version.

## Supplementary material

Supplementary Table 1 Primer sequences used in this study.

## References

[B1] Aguilera M. Martinez S. Tello M. Gallardo M. J. Garcia V. (2022). Use of cocktail of bacteriophage for *Salmonella* typhimurium control in chicken meat. Foods 11, 1164–1176. doi: 10.3390/foods11081164 PMC9029022 35454751

[B2] Ben Y. Hao J. Zhang Z. Xiong Y. Zhang C. Chang Y.. (2021). Astragaloside IV inhibits mitochondrial-dependent apoptosis of the dorsal root ganglion in diabetic peripheral neuropathy rats through modulation of the SIRT1/p53 signaling pathway. Diabetes Metab. Syndr. Obes. 14, 1647–1661. doi: 10.2147/DMSO.S301068 33883914 PMC8055373

[B3] Boels I. C. Ramos A. Kleerebezem M. De Vos W. M. (2001). Functional analysis of the lactococcus lactis *galU* and *galE* genes and their impact on sugar nucleotide and exopolysaccharide biosynthesis. Appl. Environ. Microbiol. 67, 3033–3040. doi: 10.1128/AEM.67.7.3033-3040.2001 11425718 PMC92977

[B4] Chang H. Y. Lee J. H. Deng W. L. Fu T. F. Peng H. L. (1996). Virulence and outer membrane properties of a *galU* mutant of *Klebsiella pneumoniae* CG43. Microb. Pathog. 20, 255–261. doi: 10.1006/mpat.1996.0024 8861391

[B5] Das T. Sharma S. (2018). Endophthalmitis prevention. Asia Pac J. Ophthalmol. (Phila) 7, 69–71. doi: 10.22608/APO.201866 29558571

[B6] Datsenko K. A. Wanner B. L. (2000). One-step inactivation of chromosomal genes in *Escherichia coli* K-12 using PCR products. Proc. Natl. Acad. Sci. U.S.A. 97, 6640–6645. doi: 10.1073/pnas.120163297 10829079 PMC18686

[B7] Feng S. Chen A. Wang X. Pan Z. Xu S. Yu H.. (2020). The *Glaesserella parasuis* phosphoglucomutase is partially required for lipooligosaccharide synthesis. Vet. Res. 51, 97. doi: 10.1186/s13567-020-00822-9 32736655 PMC7393335

[B8] Gomez-Baltazar A. Vazquez-Garciduenas M. S. Larsen J. Kuk-Soberanis M. E. Vazquez-Marrufo G. (2019). Comparative stress response to food preservation conditions of ST19 and ST213 genotypes of *Salmonella enterica* serotype typhimurium. Food Microbiol. 82, 303–315. doi: 10.1016/j.fm.2019.03.010 31027788

[B9] Guo R. Jiao Y. Li Z. Zhu S. Fei X. Geng S.. (2017). Safety, protective immunity, and DIVA capability of a rough mutant *Salmonella* pullorum vaccine candidate in broilers. Front. Microbiol. 8, 547. doi: 10.3389/fmicb.2017.00547 28424675 PMC5380749

[B10] Guo R. Li Z. Zhou X. Huang C. Hu Y. Geng S.. (2019). Induction of arthritis in chickens by infection with novel virulent *Salmonella* pullorum strains. Vet. Microbiol. 228, 165–172. doi: 10.1016/j.vetmic.2018.11.032 30593363

[B11] Huang K. Herrero-Fresno A. Thofner I. Skov S. Olsen J. E. (2019). Interaction differences of the avian host-specific *Salmonella enterica* serovar gallinarum, the host-generalist *S.* typhimurium, and the cattle host-adapted s. Dublin with chicken primary macrophage. Infect. Immun. 87, 1–15. doi: 10.1128/IAI.00552-19 PMC6867851 31548317

[B12] Jayakar H. R. Parvathareddy J. Fitzpatrick E. A. Bina X. R. Bina J. E. Re F.. (2011). A *galU* mutant of francisella tularensis is attenuated for virulence in a murine pulmonary model of tularemia. BMC Microbiol. 11, 179. doi: 10.1186/1471-2180-11-179 21819572 PMC3173336

[B13] Komeda Y. Icho T. Iino T. (1977). Effects of *galU* mutation on flagellar formation in *Escherichia coli* . J. Bacteriol 129, 908–915. doi: 10.1128/jb.129.2.908-915.1977 320195 PMC235029

[B14] Kovach M. E. Elzer P. H. Hill D. S. Robertson G. T. Farris M. A. Roop R. M. 2nd. (1995). Four new derivatives of the broad-host-range cloning vector pBBR1MCS, carrying different antibiotic-resistance cassettes. Gene 166, 175–176. doi: 10.1016/0378-1119(95)00584-1 8529885

[B15] Lacharme-Lora L. Owen S. V. Blundell R. Canals R. Wenner N. Perez-Sepulveda B.. (2019). The use of chicken and insect infection models to assess the virulence of African *Salmonella* typhimurium ST313. PloS Negl. Trop. Dis. 13, e0007540. doi: 10.1371/journal.pntd.0007540 31348776 PMC6685681

[B16] Meyer C. Hoffmann C. Haas R. Schubert S. (2015). The role of the *galU* gene of uropathogenic *Escherichia coli* in modulating macrophage TNF-α response. Int. J. Med. Microbiol. 305, 893–901. doi: 10.1016/j.ijmm.2015.09.004 26481693

[B17] Mills S. D. Finlay B. B. (1994). Comparison of *Salmonella* typhi and *Salmonella* typhimurium invasion, intracellular growth and localization in cultured human epithelial cells. Microb. Pathog. 17, 409–423. doi: 10.1006/mpat.1994.1086 7752882

[B18] Mollerach M. García E. (2000). The *galU* gene of *Streptococcus pneumoniae* that codes for a UDP-glucose pyrophosphorylase is highly polymorphic and suitable for molecular typing and phylogenetic studies. Gene 260, 77–86. doi: 10.1016/S0378-1119(00)00468-6 11137293

[B19] Nakamura K. Fujiki J. Nakamura T. Furusawa T. Gondaira S. Usui M.. (2021). Fluctuating bacteriophage-induced *galU* deficiency region is involved in trade-off effects on the phage and fluoroquinolone sensitivity in *Pseudomonas aeruginosa* . Virus Res. 306, 198596. doi: 10.1016/j.virusres.2021.198596 34648885

[B20] Nesper J. Lauriano C. M. Klose K. E. Kapfhammer D. Kraiss A. Reidl J. (2001). Characterization of vibrio cholerae O1 El tor *galU* and *galE* mutants: influence on lipopolysaccharide structure, colonization, and biofilm formation. Infect. Immun. 69, 435–445. doi: 10.1128/IAI.69.1.435-445.2001 11119535 PMC97901

[B21] Priebe G. P. Dean C. R. Zaidi T. Meluleni G. J. Coleman F. T. Coutinho Y. S.. (2004). The *galU* gene of *Pseudomonas aeruginosa* is required for corneal infection and efficient systemic spread following pneumonia but not for infection confined to the lung. Infect. Immun. 72, 4224–4232. doi: 10.1128/IAI.72.7.4224-4232.2004 15213167 PMC427465

[B22] Sandlin R. C. Lampel K. A. Keasler S. P. Goldberg M. B. Stolzer A. L. Maurelli A. T. (1995). Avirulence of rough mutants of *Shigella flexneri*: requirement of O antigen for correct unipolar localization of *IcsA* in the bacterial outer membrane. Infect. Immun. 63, 229–237. doi: 10.1128/iai.63.1.229-237.1995 7528731 PMC172982

[B23] Shu F. L. Jin L. Y. Liu H. Tao Z. Yin F. Xie J. S.. (2022). The *galU* gene is required for *in vivo* survival of pseudomonas plecoglossicida in large yellow croaker (Larimichthys crocea). J. Fish Dis. 46, 229–238. doi: 10.1111/jfd.13737 36484113

[B24] Vilches S. Canals R. Wilhelms M. Saló M. T. Knirel Y. A. Vinogradov E.. (2007). Mesophilic aeromonas UDP-glucose pyrophosphorylase (*GalU*) mutants show two types of lipopolysaccharide structures and reduced virulence. Microbiology 153, 2393–2404. doi: 10.1099/mic.0.2007/006437-0 17660404

[B25] Walport M. J. (2001). Complement. first of two parts. N Engl. J. Med. 344, 1058–1066. doi: 10.1056/NEJM200104053441406 11287977

[B26] Xie F. Li G. Zhang W. Zhang Y. Zhou L. Liu S.. (2016). Outer membrane lipoprotein VacJ is required for the membrane integrity, serum resistance and biofilm formation of *Actinobacillus pleuropneumoniae* . Vet. Microbiol. 183, 1–8. doi: 10.1016/j.vetmic.2015.11.021 26790928

[B27] Yang D. Huang W. Y. Li Y. Q. Chen S. Y. Su S. Y. Gao Y.. (2022). Acute and subchronic toxicity studies of rhein in immature and d-galactose-induced aged mice and its potential hepatotoxicity mechanisms. Drug Chem. Toxicol. 45, 1119–1130. doi: 10.1080/01480545.2020.1809670 32842782

[B28] Zhang Z. Du W. Wang M. Li Y. Su S. Wu T.. (2020). Contribution of the colicin receptor *CirA* to biofilm formation, antibotic resistance, and pathogenicity of *Salmonella* enteritidis. J. Basic Microbiol. 60, 72–81. doi: 10.1002/jobm.201900418 31737922

[B29] Zhao X. Yang F. Wang Y. Zhang Y. (2021). Hns mRNA downregulates the expression of *galU* and attenuates the motility of *Salmonella enterica* serovar typhi. Int. J. Med. Microbiol. 311, 151525. doi: 10.1016/j.ijmm.2021.151525 34340061

[B30] Zhou X. Li S. G. Wang J. Z. Huang J. L. Zhou H. J. Chen J. H.. (2014). Emergence of human babesiosis along the border of China with Myanmar: detection by PCR and confirmation by sequencing. Emerg. Microbes Infect. 3, e55. doi: 10.1038/emi.2014.55 26038750 PMC4150284

[B31] Zou Y. Feng S. Xu C. Zhang B. Zhou S. Zhang L.. (2013). The role of *galU* and *galE* of *Haemophilus parasuis* SC096 in serum resistance and biofilm formation. Vet. Microbiol. 162, 278–284. doi: 10.1016/j.vetmic.2012.08.006 22981816

