# Supplementary material for: Contribution of GalU to biofilm formation, motility, antibiotic and serum resistance, and pathogenicity of Salmonella Typhimurium
Source: Front Cell Infect Microbiol. 2023 Mar 20;13:1149541. doi: 10.3389/fcimb.2023.1149541 (PMC10067927; doi:10.3389/fcimb.2023.1149541)
Supplement: Supplementary Table 1 — Primer sequences used in this study. [file Table_1.docx]

**Supplementary Table:**

Table S1. Primer sequences used in this study

| Genes | primer sequences（5'→3'） | length（bp） |
| --- | --- | --- |
| *galU*-Q | F: ACCAGCGCTTCTGTTAAAAAATTTATAACGTATCGTTA | 1567 |
|  | CTTCTTAATACCCATATGAATATCCTCCTTAGTTCCTATTC |  |
| *galU-Kn* | R: TACAGATTAATGAACACGTTCAATACATGAACAG | 1786 |
|  | TCCAGGAGAATTTTAAGAGCTGCTTCGAAGTTCCTA |  |
| *galU*-JD | F: CCTGATCAAGGAATAATTTACTTCCTGGATG | 393 |
|  | R: CGAGTGTTACTATCTGCGGCAG |  |
| *galU*-HF | F:ATCGATAAGCTTGATCTGATATACTGGAACACGATACAGAT | 1015 |
|  | R:CTGCAGGAATTCGATCGTATCGTTACTTCTTAATACCCATTTC |  |
| pBBR1 | F：GTGAGTTAGCTCACTCATTAGGCAC | 554 |
|  | R：CACTCATCGCAGTCGGCCTATTG |  |
